# TNF-α/TNF-R System May Represent a Crucial Mediator of Proliferative Synovitis in Hemophilia A

**DOI:** 10.3390/jcm8070939

**Published:** 2019-06-28

**Authors:** Mirko Manetti, Silvia Linari, Eloisa Romano, Irene Rosa, Christian Carulli, Massimo Innocenti, Marco Matucci-Cerinic, Lidia Ibba-Manneschi, Giancarlo Castaman, Daniela Melchiorre

**Affiliations:** 1 Department of Experimental and Clinical Medicine, Section of Anatomy and Histology, University of Florence, 50134 Florence, Italy; 2 Center for Bleeding Disorders, Careggi University Hospital, 50134 Florence, Italy; 3 Department of Experimental and Clinical Medicine, Section of Rheumatology, University of Florence, 50134 Florence, Italy; 4 First Orthopedic Clinic, Careggi University Hospital, 50134 Florence, Italy

**Keywords:** hemophilic arthropathy, TNF-α, TNF receptors, fibroblast-like synoviocytes, proliferative synovitis

## Abstract

Hemophilic arthropathy (HA) typically begins with proliferative synovitis that shares some similarities with inflammatory arthritides, in which the proinflammatory cytokine tumor necrosis factor (TNF)-α has a crucial pathogenetic role. Inappropriate release of TNF-α was shown to contribute to arthropathy development following intra-articular bleeding in hemophilic mice. Here, we were interested in determining whether systemic levels of TNF-α and synovial tissue expression of the TNF-α/TNF receptor (TNF-R) system could be increased and related to joint damage in hemophilia A patients with severe HA. Serum levels of TNF-α measured by quantitative enzyme-linked immunosorbent assay (ELISA) were significantly increased in HA patients (*n* = 67) compared to healthy controls (*n* = 20). In HA patients, elevated TNF-α levels were significantly associated with the number of hemarthroses, the grade of synovial hypertrophy, and both the clinical World Federation of Hemophilia score and ultrasound score. The expression of TNF-α, TNF-R1, and TNF-R2 was strongly increased in HA synovium (*n* = 10) compared to the non-inflamed osteoarthritis control synovium (*n* = 8), as assessed by both immunohistochemistry and Western blotting. Increased protein levels of TNF-α, TNF-R1, and TNF-R2 were retained in vitro by HA fibroblast-like synoviocytes (*n* = 6) with respect to osteoarthritis control fibroblast-like synoviocytes (*n* = 6). Stimulation with TNF-α resulted in a significant increase in HA fibroblast-like synoviocyte proliferation quantified by the water-soluble tetrazolium (WST)-1 assay, while it had no relevant effect on osteoarthritis fibroblast-like synoviocytes. Quantification of active/cleaved caspase-3 by ELISA demonstrated that TNF-α did not induce apoptosis either in HA or in osteoarthritis fibroblast-like synoviocytes. The TNF-α/TNF-R system may represent a crucial mediator of proliferative synovitis and, therefore, a new attractive target for the prevention and treatment of joint damage in HA patients. Our findings provide the groundwork for further clinical investigation of anti-TNF-α therapeutic feasibility in hemophiliacs.

## 1. Introduction

Intra-articular bleeding plays a pivotal role in the pathogenesis of hemophilic arthropathy (HA), a major clinical manifestation of hemophilia characterized by large-joint synovitis evolving into articular cartilage destruction and subchondral bone resorption that frequently results in osteopenia or osteoporosis [1,2,3,4,5,6,7]. HA shows a tendency to be more severe in hemophilia A than in hemophilia B and, depending on the frequency of bleeding episodes and the severity of the related symptoms, may have a devastating impact on the patient’s quality of life [1,8]. Indeed, notwithstanding recent advances in the management of acute bleeds and the optimization of prophylactic factor replacement protocols, HA remains a major burden for hemophilia patients [8,9,10]. Therefore, a deeper deciphering of the pathogenetic mechanisms that underlie HA-related progressive joint damage and that, hopefully, may become novel targets for therapeutic purposes still represents a major unmet need in this area [8,9,11,12]. In HA patients, recurring bleeds into the joint cavity with tissue hemosiderin deposition are proposed to elicit synovial inflammation and hyperplasia similar to those found in inflammatory arthritides [3,4,8,9,13]. In fact, iron-laden synovial tissue produces various inflammatory cytokines promoting the proliferation of fibroblast-like synoviocytes, which are considered key players of joint inflammation and destruction through the aggressive invasion of the extracellular matrix and production of proinflammatory mediators and cartilage-degrading enzymes [3,4,8,9]. Studies on animal models have recently helped to shed some light on the underlying pathobiology driving hemarthrosis toward HA [14,15]. A rapid in vivo proinflammatory response characterized by both local and systemic innate inflammatory mediators has been demonstrated following acute hemarthrosis in hemophilic rats [14]. In particular, gene expression analysis revealed increased synovial fluid levels of multiple proinflammatory mediators, such as interleukin (IL)-1β, IL-6, and tumor necrosis factor (TNF)-α, while plasma analysis demonstrated significantly increased systemic levels of IL-6 [14]. Another recent study focusing on the detailed mapping of the early HA pathogenesis through a time-course study of induced hemarthrosis in hemophilic rats revealed proliferative synovitis dominated by neutrophil and macrophage infiltration that develops within hours after hemarthrosis [15]. Strikingly, articular cartilage and subchondral bone degeneration appeared to be early and parallel events, suggesting that these processes are also caused by a direct effect of blood exposure and cannot simply be considered consequences of inflammation [15].

The proinflammatory cytokine TNF-α has been extensively implicated in the pathogenesis of proliferative synovitis and bone resorption and is a major target for the treatment of inflammatory arthritis, such as rheumatoid arthritis (RA) [16,17,18]. Of note, a very recent experimental study has suggested that a crucial pathomechanism of inflammatory arthritis, namely the inappropriate release of TNF-α by the TNF-α convertase (TACE; also known as ADAM17) and its regulator, iRhom2, may have a pivotal role also in mediating HA in hemophilic FVIII-deficient mice [19]. Indeed, it was demonstrated that blood and its components are capable of activating the iRhom2/ADAM17-dependent release of TNF-α from macrophages [19]. Moreover, the induction of hemarthrosis led to increased levels of TNF-α in the affected joints of FVIII-deficient mice, and the inactivation of TNF-α or iRhom2 reduced synovial inflammation and osteopenia in this mouse model for HA [19]. As far as human HA is concerned, to our knowledge, the TNF-α/TNF receptor (TNF-R) system has not been specifically investigated apart from two former studies reporting increased levels of TNF-α in supernatants of cultured hemosiderotic synovial tissue from hemophilia patients [20,21]. Since the aforementioned data indicate that abnormal TNF-α release may represent a potential new target to prevent bone resorption following a joint bleed in mice, we aimed at determining whether systemic levels of TNF-α and synovial tissue expression of this proinflammatory cytokine and its receptors could be increased and related to the development of proliferative synovitis in hemophilia A patients with severe HA.

## 2. Materials and Methods

### 2.1. Patients

Sixty-seven hemophilia A patients attending the Center for Bleeding Disorders of the Careggi University Hospital in Florence, Italy, were consecutively enrolled in the study. All patients had suffered from at least one joint bleeding. Clinicodemographic characteristics and imaging findings of the study population are shown in Table 1. All patients gave informed consent, and the study was conducted in agreement with the Declaration of Helsinki and approved by the institutional review board at the Careggi University Hospital, Florence, Italy (study code no. 996, 27/12/2006).

The median age of patients was 36.3 years (range 16–69 years). All patients (100%) had severe hemophilia A (FVIII:C <1 IU/dL). Thirty-seven out of 67 patients (55.2%) were treated on demand, eight of 67 (12.0%) with primary and secondary prophylaxis, and twenty-two of 67 (32.8%) with tertiary prophylaxis. According to the last guidelines for the management of hemophilia of the World Federation of Hemophilia (WFH) [22], primary prophylaxis treatment started before the second large joint bleed and at the age of three years in the absence of osteochondral joint disease, documented by physical examination and imaging studies. Secondary prophylaxis started after two or more bleedings into large joints and before the onset of joint disease documented by physical examination and imaging studies [22]. Tertiary prophylaxis instead is when treatment started in the presence of documented joint disease. As far as viral infections are concerned, all patients were screened for HCV-RNA, anti-HCV, and HIV positivity (Table 1).

### 2.2. Clinical and Imaging Score

The severity of HA was measured using the WFH orthopedic joint scale score consisting of a physical examination and pain scale (referred to as the clinical WFH score) [23]. All patients were studied by knee X-ray and ultrasound (US). X-ray score (radiographic Pettersson score) evaluates osteoporosis, enlarged epiphysis, irregular subchondral bone surface, narrowing of the joint space, subchondral cyst formation, erosions of the joint margins, the gross incongruence of articulating bone ends, and deformity (angulation and displacement between the articulating bones) [24]. The X-ray score for a single joint varies between 0 (normal joint) and 13 (i.e., a totally destroyed joint). Knee US was performed by an experienced sonographer (DM, unaware of the severity of the clinical history), and US findings were scored according to the already published criteria (US score range 0–21 for a single joint with a cut-off less than 5) [25,26,27]. Patients were divided into three groups according to the total number of hemarthroses in their life: (1) patients with less than 10 hemarthroses (<10); (2) patients with hemarthroses 10–50 (10–50); and (3) patients with hemarthroses greater than 50 (>50) (Table 1) [26].

### 2.3. Serum TNF-α Measurements

Fresh peripheral venous blood from 67 HA patients and 20 healthy controls (median age 37.1 years, range 18–62 years) was drawn, allowed to clot for 30 min before centrifugation at 1500× *g* for 15 min. Serum samples were collected and stored in aliquots at −80 °C until used. Serum levels of TNF-α were measured with a commercially available enzyme-linked immunosorbent assay (ELISA) kit (Human TNF-alpha Quantikine HS ELISA; catalog no. HSTA00E; R&D Systems, Minneapolis, MN, USA), following the manufacturer’s protocol. Optical density was measured by a microtitre plate reader at 450 nm. The serum levels of TNF-α were read off from a standard curve according to the manufacturer’s instructions. The detection range and the sensitivity of the assay were 0.2–10 pg/mL and 0.049 pg/mL, respectively. Each sample was measured in duplicate. The inter-assay and intra-assay variances were <10%.

### 2.4. Synovial Biopsy Samples

Synovial tissue samples were obtained from 10 HA patients with severe knee arthropathy (median age 38.2 years, range 25–66 years; all treated on demand) who underwent arthroplasty during surgery as described elsewhere [28]. Synovial samples from eight male patients with degenerative knee osteoarthritis (OA) (median age 54.7 years, range 37–75 years) who underwent total joint replacement were used as controls [28]. Each synovial specimen was divided into different parts. One part was cut into small pieces, fixed in 10% buffered formalin and, after standard processing, embedded in paraffin wax and used for routine histopathological and immunohistochemical analyses. Another part was immediately frozen in liquid nitrogen for protein extraction and Western blotting analysis. The last part of the synovial tissue was used for the isolation of fibroblast-like synoviocytes.

### 2.5. Cell Isolation and Culture

Fibroblast-like synoviocytes were successfully isolated from 6 HA and 6 OA control synovial biopsy specimens subjected to a mild proteolytic treatment with 0.05% trypsin and 0.5 mM EDTA in phosphate-buffered saline for 10 min at 37 °C under gentle shaking as described elsewhere [29]. Trypsin was then neutralized with fetal calf serum (FCS) (Celbio, Milan, Italy) and cells were plated in culture dishes in RPMI 1640 medium (Cambrex Bio Science, Milan, Italy) supplemented with 15% FCS, 2 mM glutamine and penicillin-streptomycin (Cambrex Bio Science). The cells were considered fibroblast-like synoviocytes (type B synoviocytes) if negative on immunostaining with anti-CD68, anti-CD14, anti-CD11b, and anti-CD11c antibodies (markers of type A macrophage-like synoviocytes), positive by staining for the enzyme uridine diphosphoglucose dehydrogenase and if they had a spindle-shaped, fibroblast-like morphology (>95% cell purity). HA and OA fibroblast-like synoviocyte monolayers were used within the seventh passage in culture.

### 2.6. Immunohistochemistry

Synovial tissue sections (5 μm thick) were deparaffinized, rehydrated, boiled for 10 min in sodium citrate buffer (10 mM, pH 6.0) for antigen retrieval and subsequently treated with 3% H_2_O_2_ in methanol for 15 min to block endogenous peroxidase activity and subjected to immunoperoxidase-based immunohistochemistry using the ready-to-use UltraVision™ Large Volume Detection System Anti-Polyvalent, HRP kit (catalog no. TP-125-HL; Lab Vision, Fremont, CA, USA). After blocking non-specific antibody binding sites, tissue sections were incubated overnight at 4 °C with the following primary antibodies: rabbit polyclonal anti-TNF-α (1:100; catalog no. ab6671; Abcam, Cambridge, UK), rabbit polyclonal anti-TNF receptor 1 (TNF-R1) (1:500; catalog no. ab19139; Abcam) and rabbit polyclonal anti-TNF receptor 2 (TNF-R2) (1:50; catalog no. ab15563; Abcam). Isotype- and concentration-matched irrelevant rabbit IgG (Sigma-Aldrich, St. Louis, MO, USA) were used as negative controls. The day after, tissue slides were incubated sequentially with biotinylated secondary antibodies and streptavidin-peroxidase solution (both from Lab Vision) followed by immunoreactivity development using 3,3′-diaminobenzidine tetrahydrochloride (DAB; catalog no. SK-4100; Vector Laboratories, Burlingame, CA, USA) or ImmPACT VIP (catalog no. SK-4605; Vector Laboratories) peroxidase substrates as chromogens. Immunostained tissue sections were examined with a Leica DM4000 B microscope equipped with a Leica DFC310 FX 1.4-megapixel digital color camera and the Leica software application suite LAS V3.8 (Leica Microsystems, Mannheim, Germany).

### 2.7. Western Blotting

Proteins were extracted from synovial specimens by homogenization for 5 min in ice-cold lysis buffer [50 mM Tris HCl (pH 7.4), 150 mM NaCl, 1 mM EDTA, 1% Triton X-100, 0.25% sodium dodecyl sulfate (SDS)] supplemented with the protease inhibitor cocktail (Complete; Roche, Mannheim, Germany), followed by sonication. Proteins were also extracted from cultured fibroblast-like synoviocytes at basal conditions or after treatment for 24 h with recombinant human TNF-α (10 ng/mL; PeproTech, Rocky Hill, NJ, USA). Protein lysates were cleared by centrifugation for 30 min at 4 °C at 15000 rpm and assayed for protein content using Bradford’s method. Fifty micrograms of proteins were electrophoresed in SDS 12%–13.5% polyacrylamide gel under reducing conditions and then blotted to a nitrocellulose transfer membrane (Amersham Biosciences, Piscataway, NJ, USA). The membranes were blocked in 5% non-fat dry milk with 0.05% Tween-20 in phosphate buffered saline for 1 h at room temperature, and then incubated overnight at 4 °C with the following rabbit polyclonal antibodies: anti-TNF-α, anti-TNF-R1, and anti-TNF-R2 (all 1:1000; Abcam). After incubation with horseradish peroxidase-conjugated anti-rabbit IgG (Cell Signaling Technology, Beverly, MA, USA) for 1 h at room temperature, immune complexes were detected with the enhanced chemiluminescence detection system and the membranes were exposed to autoradiographic films (Amersham Biosciences). The membranes were stripped and reprobed with rabbit monoclonal anti-α-tubulin antibodies (1:1000; catalog no. #2144; Cell Signaling Technology) as the loading control. Densitometric analysis of the bands was performed using the free-share ImageJ software 64-bit Java 1.8.0_112 Windows version (NIH, Bethesda, Maryland, USA; online at http://rsbweb.nih.gov/ij) and the values were normalized to α-tubulin.

### 2.8. Cell Viability Assay

HA and OA fibroblast-like synoviocytes were seeded onto 96-well plates (40 × 10^3^ cells per well) in RPMI 1640 with 15% FCS and were left to adhere overnight. Cells were then washed three times with serum-free medium and incubated in RPMI 1640 with 0.2% FCS for an additional 24 h. Fibroblast-like synoviocytes were incubated for 24 h in RPMI 1640 with 0.2% FCS containing 10 ng/mL recombinant human TNF-α (PeproTech). Cell viability was determined by a cell proliferation assay using the water-soluble tetrazolium (WST)-1 (4-[3-(4-iodophenyl)-2-(4-nitrophenyl)-2H-5-tetrazolio]-1,3-benzene disulfonate) reagent (Roche Diagnostics, Mannheim, Germany) according to the manufacturer’s protocol. All measurements were performed in triplicate, and the results were expressed as the percentage increase/decrease in cell viability over the basal response.

### 2.9. Active Caspase-3 Assay

HA and OA fibroblast-like synoviocytes were plated in culture dishes at a concentration of 1–1.5 × 10^6^ per dish in RPMI 1640 with 15% FCS. Once at 70% confluence, fibroblast-like synoviocytes were washed three times with serum-free medium and incubated in RPMI 1640 with 0.2% FCS for 24 h. Fibroblast-like synoviocytes were then incubated for 24 h in RPMI 1640 with 0.2% FCS containing 10 ng/mL recombinant human TNF-α (PeproTech). Positive apoptosis controls were obtained by incubation of cells with staurosporine (Sigma-Aldrich). Cleaved (active) caspase-3 levels in fibroblast-like synoviocyte lysates were determined with a specific quantitative colorimetric sandwich ELISA kit designed to specifically detect human active caspase-3 protein (Invitrogen, Carlsbad, CA, USA). Fibroblast-like synoviocytes were collected, centrifuged, and then the pellet was incubated in ice-cold cell extraction buffer for 30 min and treated according to the manufacturer’s instructions. All measurements were performed in triplicate.

### 2.10. Statistical Analysis

Statistical analyses were performed using the SPSS software for Windows, version 25.0 (Statistical Package for Social Sciences Inc., Chicago, IL, USA). Data are expressed as the mean ± standard error of the mean (SEM) or median and interquartile range (IQR), as appropriate. Student’s *t*-test was used to compare two independent groups for normally distributed parameters, while the Mann–Whitney U-test was used to compare two independent groups for non-normally distributed parameters. Spearman’s rank correlation coefficient (ρ) was used to analyze the relationship between two continuous variables. Values of *p* < 0.05 were considered statistically significant.

## 3. Results

### 3.1. Circulating Levels of TNF-α Are Raised and Correlate with Joint Disease Severity in HA Patients

**Figure 1:**
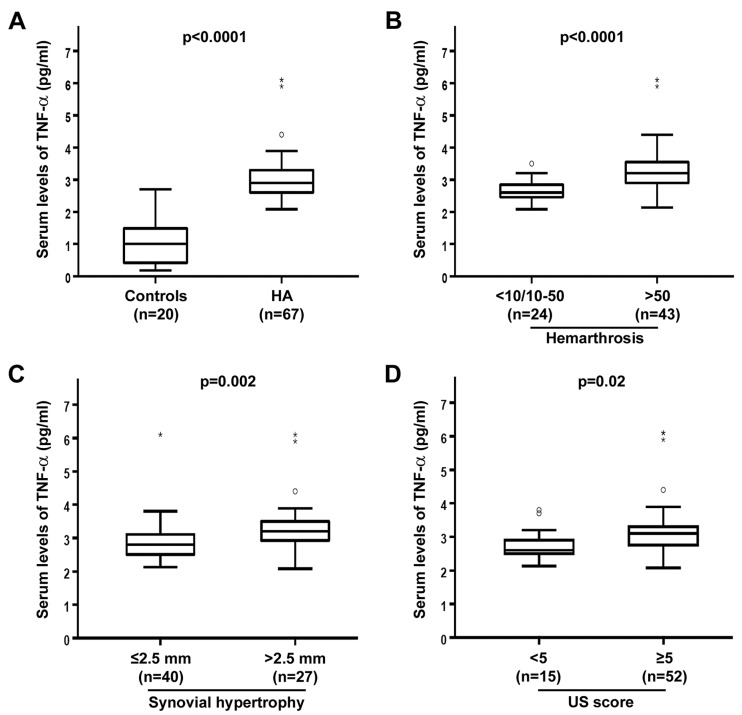
Increased circulating levels of tumor necrosis factor (TNF)-α in hemophilic arthropathy (HA). Serum concentrations of TNF-α were determined by colorimetric sandwich enzyme-linked immunosorbent assay in 67 patients with HA and 20 healthy controls. (**A**) Serum TNF-α levels in total HA patients and controls. (**B**–**D**) Serum TNF-α levels in HA patients stratified according to the number of hemarthroses (**B**), synovial hypertrophy (**C**), and ultrasound (US) score values (**D**). Boxes show 25th and 75th percentiles. Vertical lines below and above boxes show 10th and 90th percentiles. Lines inside the boxes represent the medians, circles the outliers, and asterisks the extreme values. Significant differences between groups are indicated. Mann–Whitney U-test was used for statistical analysis.

TNF-α was measured in serum samples from HA patients and healthy controls by quantitative sandwich ELISA. Serum levels of TNF-α were significantly increased in HA patients (median 2.9 pg/mL, IQR 2.6–3.3 pg/mL) compared to healthy controls (median 1.0 pg/mL, IQR 0.4–1.5 pg/mL) (*p* < 0.0001) (Figure 1A). No significant differences in serum TNF-α levels were detected according to the HCV-RNA, anti-HCV, or HIV status of HA patients.

When HA patients were stratified according to the total number of hemarthroses in their life, there were no differences in TNF-α serum levels between the group with <10 and the group with 10–50 episodes of hemarthrosis (*n* = 7 patients and *n* = 17 patients, respectively). Therefore, these patients were grouped (*n* = 24 patients) for further comparison with the group with >50 episodes of hemarthrosis (*n* = 43 patients). Circulating levels of TNF-α in the >50 group (median 3.2 pg/mL, IQR 2.9–3.6 pg/mL) were significantly higher than in the <10/10–50 groups (median 2.6 pg/mL, IQR 2.5–2.9 pg/mL) (*p* < 0.0001) (Figure 1B). As far as synovial hypertrophy is concerned, TNF-α levels were significantly elevated in HA patients with the synovial thickness of >2.5 mm (median 3.2 pg/mL, IQR 2.9–3.5 pg/mL) compared to patients with the synovial thickness of ≤2.5 mm (median 2.8 pg/mL, IQR 2.5–3.1 pg/mL) (*p* = 0.002) (Figure 1C). Furthermore, serum TNF-α levels were significantly greater in HA patients with US score ≥5 (median 3.1 pg/mL, IQR 2.7–3.3 pg/mL) than in those with US score <5 (median 2.6 pg/mL, IQR 2.5–2.9 pg/mL) (*p* = 0.02) (Figure 1D).

**Figure 2:**
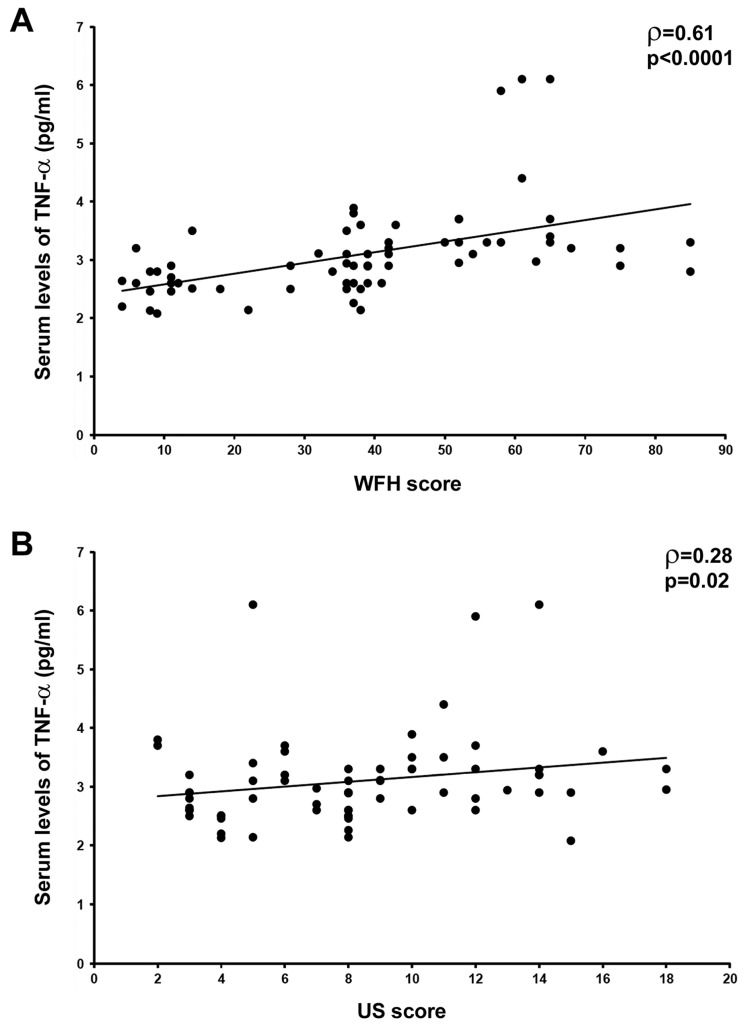
Circulating tumor necrosis factor (TNF)-α levels positively correlate with clinical World Federation of Hemophilia (WFH) score and ultrasound (US) score in patients with hemophilic arthropathy. Correlation of serum TNF-α levels with clinical WFH score (**A**) and US score (**B**). Data are shown as scatterplots, each dot representing a patient. Spearman’s rank correlation coefficient (ρ) and *p* values are indicated.

In HA patients, a significant positive correlation between the circulating levels of TNF-α and both clinical WFH score (ρ = 0.61, *p* < 0.0001) and US score (ρ = 0.28, *p* = 0.02) was found (Figure 2A,B). Moreover, we observed a trend toward a positive correlation between circulating TNF-α and radiographic Pettersson score, though this analysis did not reach statistical significance (ρ = 0.15, *p* = 0.058).

### 3.2. The Expression of TNF-α, TNF-R1, and TNF-R2 Is Strongly Increased in HA Synovial Tissue and Cultured HA Fibroblast-Like Synoviocytes

**Figure 3:**
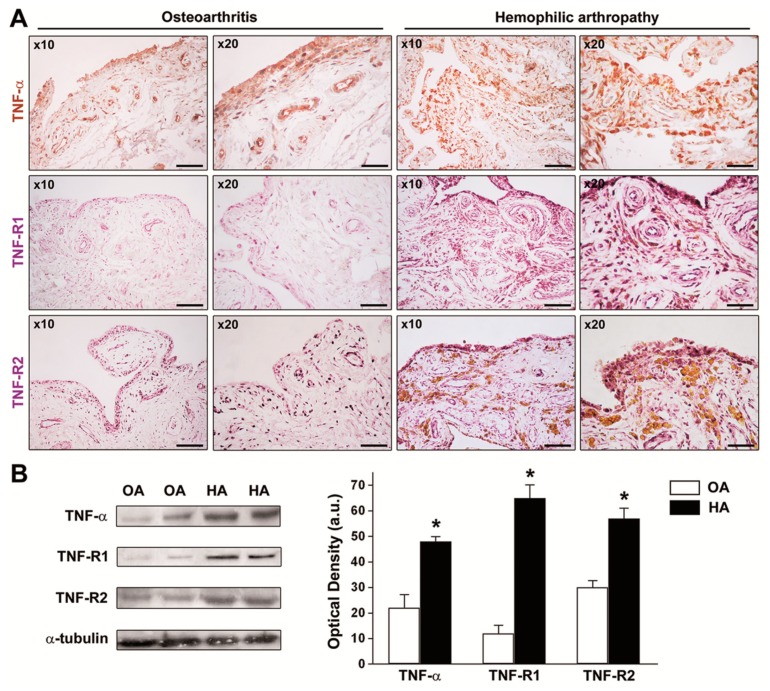
Expression of tumor necrosis factor (TNF)-α, TNF receptor 1 (TNF-R1), and TNF receptor 2 (TNF-R2) in synovial tissue from patients with hemophilic arthropathy (HA) and osteoarthritis (OA). (**A**) Representative microphotographs of tissue sections subjected to immunoperoxidase-based immunohistochemical staining for TNF-α (brown color), TNF-R1, and TNF-R2 (purple color) are shown. TNF-α, TNF-R1, and TNF-R2 immunostaining is strongly increased either in the hyperplastic lining or in the sublining layers of the HA synovium compared to non-inflamed OA control synovium. Original magnification: ×10 and ×20. Scale bar: 200 µm (×10 panels), 100 µm (×20 panels). (**B**) Western blotting of total protein extracts from the synovium of HA patients (*n* = 10) and OA controls (*n* = 8). Representative immunoblots for TNF-α, TNF-R1, and TNF-R2 are shown. The densitometric analysis of the bands normalized to α-tubulin is reported in the histograms. Data are the mean ± SEM of the optical density in arbitrary units (a.u.). * *p* < 0.05 vs. OA (Student’s *t*-test).

The ex vivo expression of TNF-α, TNF-R1, and TNF-R2 in knee synovial tissue sections from HA patients and OA controls was investigated by immunoperoxidase-based immunohistochemical staining procedures. TNF-α, TNF-R1, and TNF-R2 immunostainings were strongly increased either in the hyperplastic synovial lining layer or the synovial sublining layer of HA compared to non-inflamed OA control synovial specimens, particularly in synoviocytes, the vascular endothelium, and perivascular cells (Figure 3A). As displayed in Figure 3B, the analysis of protein levels in tissue homogenates by Western blotting confirmed that the expression of TNF-α, TNF-R1, and TNF-R2 was significantly increased in HA synovium compared with OA control synovium (*p* < 0.05 for all comparisons).

**Figure 4:**
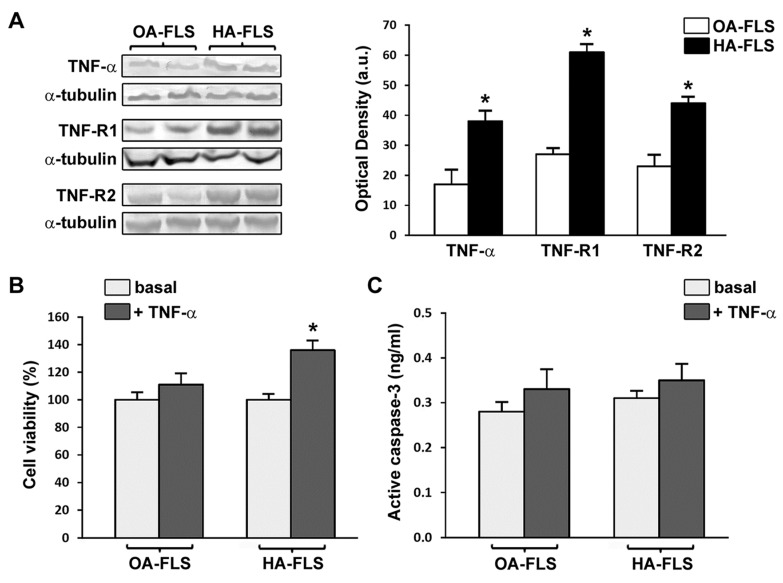
Fibroblast-like synoviocytes (FLS) from patients with hemophilic arthropathy (HA-FLS) overexpress tumor necrosis factor (TNF)-α, TNF receptor 1 (TNF-R1), and TNF receptor 2 (TNF-R2) and effectively proliferate in response to TNF-α. (**A**) Western blotting of total protein extracts from cultured HA-FLS and osteoarthritis control FLS (OA-FLS). Representative TNF-α, TNF-R1, and TNF-R2 immunoblots are shown; α-tubulin was used as a loading control. The densitometric analysis of the bands normalized to α-tubulin is reported in the histograms. Data are mean ± SEM of optical density in arbitrary units (a.u.). * *p* < 0.05 vs. OA-FLS (Student’s *t*-test). (**B**) Cell viability evaluated at the basal condition or after treatment with recombinant human TNF-α (10 ng/mL) using the water-soluble tetrazolium (WST)-1 cell proliferation reagent. Cell viability in response to TNF-α is expressed as the percentage increase/decrease over the basal response for both HA-FLS and OA-FLS. Bars represent the mean ± SEM. Results are representative of three independent experiments performed with each one of the six HA-FLS and six OA-FLS lines. * *p* < 0.05 vs. the respective basal condition (Student’s *t*-test). (**C**) Levels of active (cleaved) caspase-3 in HA-FLS and OA-FLS, as measured by the specific enzyme-linked immunosorbent assay on cell lysates. Data are the mean ± SEM of three independent experiments performed in triplicate with each one of the six HA-FLS and six OA-FLS lines.

Elevated protein expression levels of TNF-α, TNF-R1, and TNF-R2 were retained in vitro by cultured HA fibroblast-like synoviocytes (*p* < 0.05 vs. OA fibroblast-like synoviocytes for all comparisons) (Figure 4A).

### 3.3. TNF-α Fosters HA Fibroblast-Like Synoviocyte Proliferation

We evaluated the ability of TNF-α to influence fibroblast-like synoviocyte proliferation by WST-1 cell viability assay. Stimulation with recombinant human TNF-α (10 ng/mL) resulted in a significant increase in the proliferation of HA fibroblast-like synoviocytes (*p* < 0.05 vs. the basal condition) (Figure 4B). Conversely, TNF-α had no relevant effect on proliferation of OA control fibroblast-like synoviocytes (Figure 4B). Quantification of active/cleaved caspase-3 levels demonstrated that TNF-α did not induce apoptosis either in HA or in OA fibroblast-like synoviocytes (Figure 4C).

## 4. Discussion

This study was undertaken based on the evidence that (i) HA usually begins with proliferative synovitis that shares some similarities with inflammatory arthritis in which the TNF-α cytokine is recognized as a crucial pathogenetic orchestrator, and (ii) inappropriate release of TNF-α contributes to arthropathy development following intra-articular bleeding in hemophilic FVIII-deficient mice [3,4,8,9,19]. To investigate whether the TNF-α/TNF-R pathway could contribute to the pathogenesis of human HA, here we combined ex vivo studies on serum samples and synovial biopsies and in vitro studies on isolated fibroblast-like synoviocytes from hemophilia A patients with severe HA. 

The findings that systemic levels of TNF-α were strongly increased and correlated with disease severity in our patients indicate that this cytokine may be tightly related to the evolution of HA. Indeed, we found significant associations between circulating levels of TNF-α and multiple clinical parameters of HA, including the number of hemarthroses, the grade of synovial hypertrophy, and the clinical WFH score and US score, which altogether suggest a role for TNF-α in the progression of blood-induced arthropathy and its possible suitability in the future as a biomarker of disease severity that could potentially be used to identify patients with progressing joint disease. Moreover, our observation that local synovial expression of TNF-α, TNF-R1, and TNF-R2 is robustly increased in HA and retained in vitro by cultured HA fibroblast-like synoviocytes provides strong support to the notion that TNF-α/TNF-R signaling may be dysregulated in hemophiliacs as previously reported in hemophilic FVIII-deficient mice [19]. This assumption is further corroborated by our in vitro findings obtained through functional assays and demonstrating that, at variance with OA control fibroblast-like synoviocytes, HA fibroblast-like synoviocytes are strongly responsive to the stimulation with TNF-α. Considering that the TNF-α/TNF-R system may activate a multitude of intracellular signaling pathways resulting in the promotion of either cell survival/proliferation or apoptosis in different contexts [30,31], we tested the possible effects of TNF-α challenge on both cellular activities. Hence, we found that TNF-α is capable of greatly fostering the proliferation of HA fibroblast-like synoviocytes, while it did not promote cell death signals as testified by unchanged levels of active/cleaved caspase-3 compared with resting cells. Taken together, our findings support the concept that TNF-α may represent an important mediator of proliferative synovitis in patients with severe HA. Interestingly, this evidence further supports that HA may share important cellular and molecular pathomechanisms with RA [4,8,29], as it is well known that TNF-α induces sustained signaling and a prolonged and unremitting inflammatory response in RA synovial fibroblasts which orchestrates pannus formation and subsequent articular cartilage invasion and destruction [32]. 

Consistent with recent observations in a mouse model for HA [19], the data from HA patients presented herein open a new scenario putting the proinflammatory TNF-α pathway among those worth to be targeted for therapeutic purposes. The feasibility of TNF-α antagonism as a novel strategy to treat HA clearly deserves a thorough investigation, especially if considering that, over the last two decades, anti-TNF-α biologics became the most powerful tools for controlling patients suffering from a number of rheumatic diseases, such as RA, psoriatic arthritis, and ankylosing spondylitis, with sustained efficacy and acceptable safety profiles as demonstrated in long-term follow-up studies [16,33,34]. In a small case series of hemophilia A patients with overlapping RA or psoriatic arthritis, systemic anti-TNF-α therapy with adalimumab proved to be useful not only in controlling synovitis and inducing disease remission but also in reducing joint bleeding [35]. Of note, through careful infectious screening and serological monitoring, anti-TNF-α therapy could be safely used even in hemophiliacs presenting viral infections, which are usually considered a major contraindication for this kind of treatment [35]. Furthermore, in hemophilic mice subjected to intra-articular bleeding, treatment with etanercept to block TNF-α reduced synovial inflammation and largely prevented the activation of osteoclasts and the resulting osteopenia in the trabecular bone adjacent to the hemarthrosis [19]. It is worth noting that the lack of osteopenia in the contralateral uninjured joint led the authors to propose that, in this mouse model of HA, the release of TNF-α from the hemarthrosis acts locally and not systemically, which might suggest the use of an intra-articular anti-TNF-α biologic administration [19]. However, the clinical evidence that HA may often affect multiple joints in the same patient and our current observation that circulating TNF-α is strongly increased and correlated with HA severity in hemophilia A patients might, instead, be in favor of a systemic anti-TNF-α therapeutic approach. Indeed, the significant relationship between serum levels of TNF-α and the number of hemarthroses in our patients supports the concept that joint bleeds, especially repetitive bleeds, might promote a systemic proinflammatory state.

Although our novel findings highlight a non-negligible participation of the TNF-α/TNF-R system to the pathobiology of human HA, we are aware that further in-depth molecular studies will be required to better decipher the mechanisms of action of this pleiotropic pathway and to identify potential downstream therapeutic targets. In fact, it is well known that TNF-α activates complex signaling cascades and induces many other proinflammatory mediators, such as IL-6, whose signaling is currently attracting much attention as a target for the treatment of chronic arthritis and, possibly, also to protect against HA [36,37,38,39]. Whether abnormal iRhom2/ADAM17-dependent membrane-anchored TNF-α precursor shedding from macrophages might be primarily responsible for the increased levels of TNF-α in HA patients, as demonstrated in hemophilic mice [19], also remains to be determined. Although a previous important study reported that TNF-α is not pivotal in blood-induced cartilage damage [40], our in vitro data suggest an important role for this cytokine at least in the induction of HA fibroblast-like synoviocyte proliferation and synovial hyperplasia, and further investigations are necessary to elucidate its possible participation in additional pathomechanisms of HA, such as neoangiogenesis [41,42]. Finally, in line with recent findings suggesting that HA in hemophilia B may be less severe than in hemophilia A as reflected by either clinical parameters or differences in levels of TNF/TNF-R superfamily members controlling bone turnover, such as osteoprotegerin and activator of nuclear factor-kB ligand [8,26], it will be of major importance to also search for possible differences in the proinflammatory TNF-α pathway between the two types of hemophilia. Owing to the cross-sectional design of our study, we consider that further prospective investigations in larger patient cohorts will be necessary to shed light on the potential of the TNF-α pathway as a biomarker reflecting HA evolution. 

In summary, our results add to recent data on murine HA [19] and provide new insights into the role of the TNF-α/TNF-R system in blood-induced proliferative synovitis, thereby strengthening the notion that this pathway might represent a new target for the prevention and treatment of joint damage in HA patients. Indeed, at present HA remains the largest cause of morbidity in patients with hemophilia despite advancements in factor replacement therapy and new developments in innovative therapeutic approaches [8,9,10]. Especially when considering that factor replacement alone may be not sufficient in advanced HA, we believe that our findings provide the groundwork for further clinical investigation of anti-TNF-α therapeutic feasibility in hemophiliacs.

## Figures and Tables

**Table 1:** Clinical characteristics and imaging findings of 67 hemophilia A patients.

Clinical characteristics/Imaging Findings	Patients
Median age (range), years	36.3 (16–69)
Primary and secondary prophylaxis treatment, *n* (%)	8 (12.0%)
Tertiary prophylaxis treatment, *n* (%)	22 (32.8%)
On demand treatment, *n* (%)	37 (55.2%)
**Viral infections,***n* (%)	
HCV-RNA	29 (43.3%)
Anti-HCV	43 (64.2%)
HIV	14 (20.9%) *
**Hemarthroses,***n* (%)	
<10	7 (10.4%)
10–50	17 (25.4%)
>50	43 (64.2%)
**Synovial hypertrophy,***n* (%)	
≤2.5 mm	40 (59.7%)
>2.5 mm	27 (40.3%)
**Clinical WFH score,** mean ± SD	37.6 ± 21.2
**RadiographicPettersson score,** mean ± SD	8.46 ± 7.62
**US score,** mean ± SD	8.32 ± 4.09
*n* (%)	
<5	15 (22.4%)
≥5	52 (77.6%)

WFH: World Federation of Hemophilia; US: ultrasound. * All HIV positive with undetectable viremia (HIV-RNA < 20 cp/mL) and receiving antiretroviral therapy.
